# Next Generation Sequencing of Tumor and Matched Plasma Samples: Identification of Somatic Variants in ctDNA From Ovarian Cancer Patients

**DOI:** 10.3389/fonc.2021.754094

**Published:** 2021-09-30

**Authors:** Ana Barbosa, Pedro Pinto, Ana Peixoto, Joana Guerra, Manuela Pinheiro, Catarina Santos, Carla Pinto, Carla Escudeiro, Carla Bartosch, Rui Santos, Andreia Brandão, João Silva, Manuel R. Teixeira

**Affiliations:** ^1^ Cancer Genetics Group, Instituto Português de Oncologia (IPO)-Porto Research Center (CI-IPOP), Portuguese Oncology Institute of Porto (IPO-Porto), Porto, Portugal; ^2^ Department of Genetics, Portuguese Oncology Institute of Porto (IPO-Porto), Porto, Portugal; ^3^ Department of Pathology, Portuguese Oncology Institute of Porto (IPO-Porto), Porto, Portugal; ^4^ Institute of Biomedical Sciences Abel Salazar (ICBAS), University of Porto, Porto, Portugal

**Keywords:** ovarian cancer, liquid biopsy, ctDNA = circulating tumor DNA, NGS - next generation sequencing, tumor hetereogeneity

## Abstract

Genetic testing to detect somatic alterations is usually performed on formalin-fixed paraffin-embedded tumor samples. However, tumor molecular profiling through ctDNA analysis may be particularly interesting with the emergence of targeted therapies for ovarian cancer (OC), mainly when tumor is not available and biopsy is not viable, also allowing representation of multiple neoplastic subclones. Using a custom panel of 27 genes, next-generation sequencing (NGS) was performed on tumor and matched plasma samples from 96 OC patients, which were combined in two groups (treatment naive and post-treatment). Overall, at least one somatic variant present in the tumor sample was also detected in the matched plasma sample in 35.6% of the patients, a percentage that increased to 69.6% of the treatment naive patients and 83.3% of those with stage IV disease, showing the potential of ctDNA analysis as an alternative to identify somatic variants in these patients, namely those that have predictive value for targeted therapy. In fact, of the two treatment-naive patients with somatic *BRCA1* variants identified in tumor samples, in one of them we detected in ctDNA a *BRCA1* somatic variant that was present in the tumor with a VAF of 53%, but not in the one that had a VAF of 5.4%. We also showed that ctDNA analysis has a complementary role to molecular unraveling of inter- and intra-tumor heterogeneity, as exemplified by one patient diagnosed with bilateral OC in which different somatic variants from both tumors were detected in ctDNA. Interestingly, as these bilateral tumors shared a rare combination of two of the three variants identified in ctDNA, we could conclude that these morphologically different tumors were clonally related and not synchronous independent neoplasias. Moreover, in the post-treatment group of patients with plasma samples collected after surgery, those with detectable somatic variants had poor prognosis when compared with patients with no detectable somatic variants, highlighting the potential of ctDNA analysis to identify patients at higher risk of recurrence. Concluding, this study demonstrated that somatic variants can be detected in plasma samples of a significant proportion of OC patients, supporting the use of NGS-based ctDNA testing for noninvasive tumor molecular profiling and to stratify patients according to prognosis.

## Introduction

Ovarian cancer (OC) is the third most common malignant disease among patients with gynecological neoplasia, being the eighth most common cancer and the eighth most common cause of cancer-related death in women (1). OC is characterized by few and unspecific symptoms, late diagnosis, and poor survival. During the last decades, several efforts have been done to improve the outcomes for OC patients, through the development of new therapies. Since 2014, the treatment of OC has been improved with the emergence of the poly ADP-ribose polymerase inhibitors (PARPi) (2–5). With the PARPi approval, BRCA status became an important predictor of response and susceptibility to this class of drugs in addition to family risk assessment. Therefore, tumor genetic testing in OC patients can provide predictive information to guide treatment with PARPi. Consequently, genetic testing (somatic and germline) for all women diagnosed with OC is recommended (6). Comprehensive genetic testing is usually performed in formalin fixed paraffin embedded (FFPE) tumor samples and blood samples are used to verify whether the variants found in tumor are germline or somatic and to look for germline exon rearrangements (7). FFPE tumor samples are often used for molecular profiling, as it is widely available and easy to use and to store. Furthermore, using these samples it is possible to define areas enriched for neoplastic cells and select these for sequencing and consequently avoiding contamination with normal tissue, which improves the sensitivity to detect somatic variants. However, the DNA extracted from FFPE samples is sometimes of poor quality and not suitable for molecular analysis (8), and may not constitute an ideal source for an accurate genetic characterization when inter- and intra-tumoral heterogeneity is present (9). Furthermore, due to its invasive nature and associated risks, it is difficult to obtain additional tumor biopsies over the course of the disease. In the context of OC, there are other challenges, such as the difficulty of performing tumor biopsy due to anatomical limitations or risk of spillage (10), so the majority of tumor samples from OC patients are obtained at the time of the surgery. However, these samples may not be the most suitable for tumor molecular profiling, as several OC patients are treated with chemotherapy before debulking surgery, which can modify the tumor genetic profile as the result of mutational shifts induced by chemotherapy (7, 11).

Circulating-cell free DNA (cfDNA) consists of small fragments of DNA that are believed to be released by the cells through apoptotic and necrotic processes (12). In cancer patients, a fraction of cfDNA is derived from tumor cells, therefore containing the associated genetic alterations, being named circulating cell-free tumor DNA (ctDNA) (13). In the last decades, several studies showed that ctDNA has a great potential as a biomarker in several types of cancer (14). The use of ctDNA, called liquid biopsy, in the management of cancer patients has several advantages as an alternative for tissue biopsies. First, liquid biopsy provides a better representation of the overall tumor genome since it comprises DNA released by tumor cells from both primary tumor and metastases, minimizing the problem of inter- and intratumor heterogeneity (15). Second, it is possible to quantify cfDNA and its concentration seems to reflect the tumor burden, making cfDNA measurements potentially useful to monitor tumor dynamics (16). Third, blood samples are minimally invasive, allowing repeated sampling and “real-time” monitoring during the entire course of the disease. On the other hand, ctDNA is highly fragmented and its fraction in circulation can be as low as 0.01%, making its detection difficult, particularly in early-stage tumors (17). Therefore, ctDNA detection requires high-sensitive approaches.

The potential of liquid biopsy in OC management has gained increasing attention and has several possible applications, including diagnosis, prognosis, evaluation of therapy response, monitoring the emergence of resistance during the course of treatment and disease relapse prediction. The vast majority of published studies in OC focused on the quantification of total levels of cfDNA in plasma or serum of OC patients (18). However, the detection of mutations in ctDNA is expected to be more specific for those applications. Here, we aimed to evaluate the sensitivity of detection of tumor somatic variants in plasma samples from OC patients by comparing next generation sequencing (NGS) of FFPE tumor samples and paired cfDNA from 96 OC patients.

## Material and Methods

### Patients and Samples

This study included 96 tumor samples and 96 matched plasma samples from 96 women diagnosed with OC, collected between 2016 and 2019 at the Portuguese Oncology Institute of Porto (IPO-Porto). The study was approved by the institutional review board (CI-IPOP-35-2016) and written informed consent was obtained from all patients. FFPE tumor samples were obtained from all patients, after evaluation by a pathologist, who delimited areas with >50% cancer cells. From each patient, peripheral blood samples were obtained for plasma collection. Twenty-three plasma samples were collected from newly diagnosed patients, before any treatment. Seven samples were collected after surgery and before treatment with chemotherapy. Twelve samples were collected at recurrence. The remaining 53 plasma samples were collected when patients had already received at least one cycle of chemotherapy, and 6 of these were collected at progression during treatment. For subsequent analysis, the samples were combined in two groups: treatment naive group and post-treatment group. The first includes patients from whom tumor and plasma samples were collected at diagnosis, before any treatment (n=23). In the second group includes those patients from whom plasma samples were obtained after treatment (n=73). All patients included in this study were previously tested for germline variants in 10 genes in a study from our group that aimed to test the yield of germline variants using FFPE samples for NGS (19). The clinicopathological features of OC patients included in the study are presented in **Table 1**.

**Table 1:** Clinical features of the 96 patients included in the study.

Clinical features		N (%)
Age, median (range years)		57 (27–80)
Figo Stage	Figo stage I	14 (14.6)
	Figo stage II	7 (7.3)
	Figo stage III	55 (57.3)
	Figo stage IV	20 (20.8)
Histological subtype	High-grade serous	72 (71.9)
	Low-grade serous	8 (8.3)
	Clear cell	6 (6.3)
	Endometrioid	7 (7.3)
	Mucinous	1 (1.0)
	Mixed	3 (3.1)
	Carcinosarcoma	2 (2.1)
Patient groups	Treatment naive group	23 (24.0)
	Post-treatment group	73 (76.0)

### DNA Extraction

DNA extraction from FFPE samples was performed using the Cobas^®^ DNA Sample Preparation Kit (Roche Diagnostics, Basel, Switzerland) according to the manufacturer’s protocol. Quality of DNA samples was measured using the Qubit^®^ Fluorometer with the Qubit dsDNA HS assay kit (Thermo Fisher Scientific, Waltham, MA, USA). Peripheral blood samples collected in BD Vacutainer K_2_EDTA (Becton Dickenson, Franklin Lakes, NJ, USA) tubes and plasma samples were obtained by centrifugation within 2 hours of the blood collection. We performed a two-step centrifugation, the first one 10 minutes at 1600g at room temperature. After centrifugation, the supernatant was collected and centrifuged 10 minutes at 6000g at room temperature, to remove remaining cells. Plasma supernatant was transferred to 1.5mL tubes and stored at -80°C until use. DNA was extracted from 3mL of plasma samples using the QIAamp circulating nucleic acid kit following the manufacturer’s protocol (QIAGEN, Antwerp, Belgium).

### Next Generation Sequencing and Bioinformatic Analysis

Next-generation sequencing (NGS) was performed in 96 tumor samples and matched plasma samples using a customized QIASeq Targeted DNA Panel containing 27 genes (QIAGEN, Antwerp, Belgium) previously described as frequently mutated in OC in the literature and COSMIC database (**Table S1**– **Supplementary Data**) (20–23). Library preparation was performed according to the manufacturer’s instructions and final libraries were quantified on a 4200 TapeStation System (Agilent Technologies Inc, Santa Clara, CA). Sequencing was carried out using a high-output kit in the NextSeq 550 System (Illumina, Inc., San Diego, CA, USA) in a 2x151 bp paired-end run. Sequencing alignment and variant calling were performed using Qiagen GeneGlobe Data Analysis Center (QIAGEN, Antwerp, Belgium). For plasma samples, sequencing alignment and variant calling was also performed using CLC Genomics Workbench 21 (CLC Bio, version 21.0.3). The resulting .vcf files were imported to GeneticistAssistant™ software (SoftGenetics, LLC, State College, PA, USA) for variant annotation. All variants with a minor allele frequency (MAF) greater than 1% and synonymous variants were excluded. For MAF filtering, data were obtained from the 1000 Genomes Project (1000G), Genome Aggregation Database (gnomAD) and Exome Aggregation Consortium (ExAC) databases. Additionally, in the analysis of tumor samples we excluded variants present in the tumor with an allele frequency (VAF) lower than 5%.

### Variant Analysis

After variant filtering, all the variants that had been described as pathogenic/likely pathogenic in ClinVar or having literature evidence supporting their pathogenicity were retained as deleterious. Nonsense, frameshift, as well as canonical splice site variants, were also retained since they are considered to have very strong evidence of pathogenicity (24). Given that the aim of this study is the analysis of detection of somatic variants in ctDNA, inframe deletion and/or insertion variants not classified were also retained. The potential pathogenicity of missense VUS was evaluated using MetaLR and MetaSVM, which combine 10 in silico prediction tools (SIFT, PolyPhen-2 HDIV, PolyPhen-2 HVAR, GERP++, MutationTaster, Mutation Assessor, FATHMM, LRT, SiPhy, and PhyloP) and the maximum minor allele frequency (MMAF) from the 1000G project (25). We also used Combined Annotation-Dependent Depletion (CADD), which is an integrative annotation built from more than 60 genomic features for scoring the deleteriousness of single nucleotide variants (SNVs) and insertion/deletion variants (26). Missense VUS were retained only if they were predicted to be damaging by MetaLR, MetaSVM, and CADD. Variants described in ClinVar as benign/likely benign were classified as benign and discarded.

### Validation of Germline and Somatic Variants

Variants present with a VAF at nearly 50% in plasma samples, suspected to be germline, were confirmed by Sanger sequencing in peripheral blood samples. Somatic variants were confirmed by Sanger sequencing in DNA extracted from FFPE samples. Sanger sequencing was performed in a 3500 Genetic Analyzer (Applied Biosystems Foster City, CA, USA), using the BigDye^®^ Terminator v3.1 Cycle Sequencing Kit (Applied Biosystems), following the manufacturer’s instructions.

## Results

### Identification of Somatic Variants in Tumor Samples

**Figure 1:**
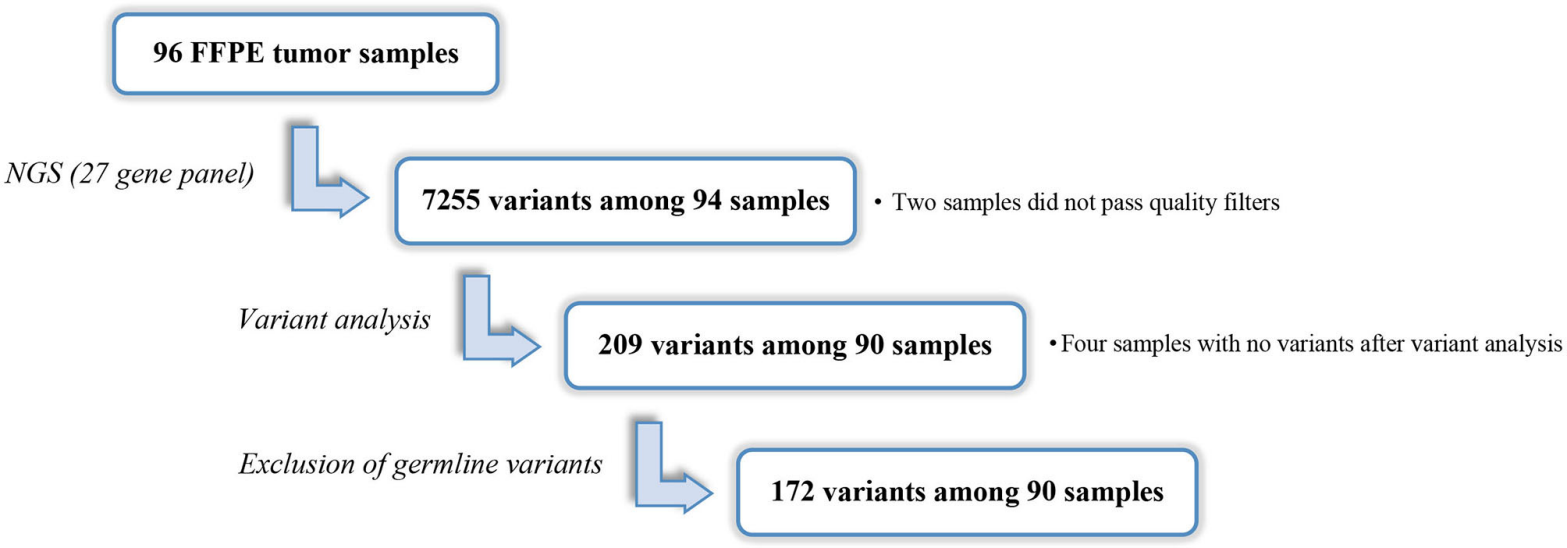
Identification of somatic variants in tumor samples.

**Figure 2:**
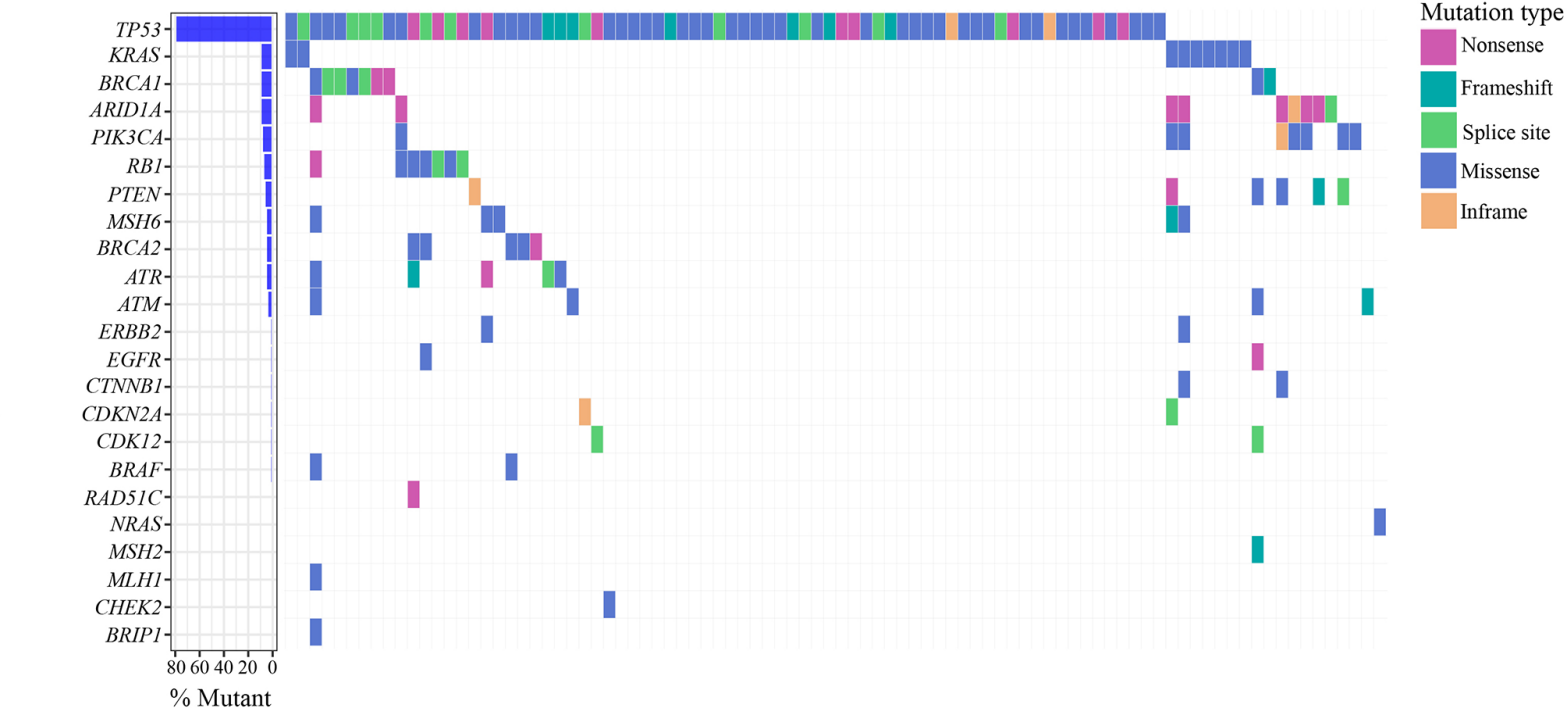
Spectrum of somatic variants identified in ovarian tumor samples.

Ninety-six FFPE samples were analyzed by NGS (mean UMI depth =1276). A total of 7255 variants were annotated and classified among all the samples with exception of cases OC65 and OC85 that did not pass the quality filters. After variant filtering, 335 variants remained, of which 99 were classified as pathogenic/likely pathogenic in ClinVar. Among variants not classified in ClinVar, 41 variants were nonsense, frameshift and variants located at canonical ± 1 or 2 splice sites and were therefore considered pathogenic (24). Eight variants were inframe deletions and/or insertions that were also retained. The remaining 187 variants were missense alterations whose potential pathogenicity was evaluated through in silico prediction tools and 61 variants predicted to have deleterious impact on the protein were retained. After variant analysis, 209 variants were retained. At least one variant per tumor sample was identified, except in four cases (OC13, OC29, OC48 and OC63), in which no variants were identified. In addition to the 26 variants identified as germline in our previous study (19), 11 variants with a VAF at nearly 50% in plasma samples were tested in peripheral blood samples and were confirmed to be germline. Excluding the 37 germline variants, we retained 172 somatic variants, among 90 patients and across 23 of the 27 genes included in the panel (**Figure 1**). *TP53* variants were identified in 72 (78.0%) of the 94 samples, being the most prevalent mutated gene, followed by *BRCA1*, *ARID1A*, and *KRAS* in which variants were found in 9 (9.6%) samples each. Variants in *PIK3CA* were detected in eight (8.5%) samples, followed by *PTEN* and *RB1* that were found mutated in six (6.4%) samples each. Variants in *BRCA2*, *ATM*, *ATR* and *MSH6* were identified in five (5.3%) samples each. For the remaining genes, a frequency of mutations equal or less than two percent was observed (**Figure 2**).

### Mutational Concordance of Tumor and Plasma Samples

We next analyzed 96 plasma samples by NGS (mean UMI depth = 513). Using Qiagen GeneGlobe Data Analysis Center, a total of 3414 variants were annotated and classified among the 96 plasma samples. After variant filtering, 239 variants remained, of which 43 were classified as pathogenic/likely pathogenic in ClinVar. Among variants not classified in ClinVar, 39 were nonsense, frameshift and variants located at canonical ± 1 or 2 splice sites, and were therefore considered pathogenic (24). Five variants were inframe deletions and/or insertions that were also retained. The remaining 150 variants were missense alterations, of which 43 were retained because they were predicted to have deleterious impact on the protein by in silico tools. After variant analysis, we retained 130 variants, of which 37 were germline variants previously identified, which were excluded. Thus, 93 somatic variants were identified in plasma samples among 51 patients, with an average of two variants in each plasma sample. The overall mutational concordance between tumor and matched plasma samples was 26.7%. Considering only the group of untreated patients, at least one somatic variant was detected in matched plasma sample in 13 patients (56.5%). The detection rate of somatic variants in plasma samples was higher in patients with stage IV disease (83.3%). In patients diagnosed with stage III and stage I disease, somatic variants were detected in plasma samples in 46.2% and 50% of the cases, respectively. Comparing with tumor testing of the post-treatment patient group, at least one somatic variant was detected in matched plasma sample in 17.9% of them. In seven patients with plasma samples collected after surgery and before treatment with chemotherapy, none of the variants identified in tumor samples were detected in the matched plasma samples. In a subgroup of 12 patients from whom plasma samples were collected during follow-up at the time of disease recurrence, somatic variants were detected in 25% of the cases.

**Figure 3:**
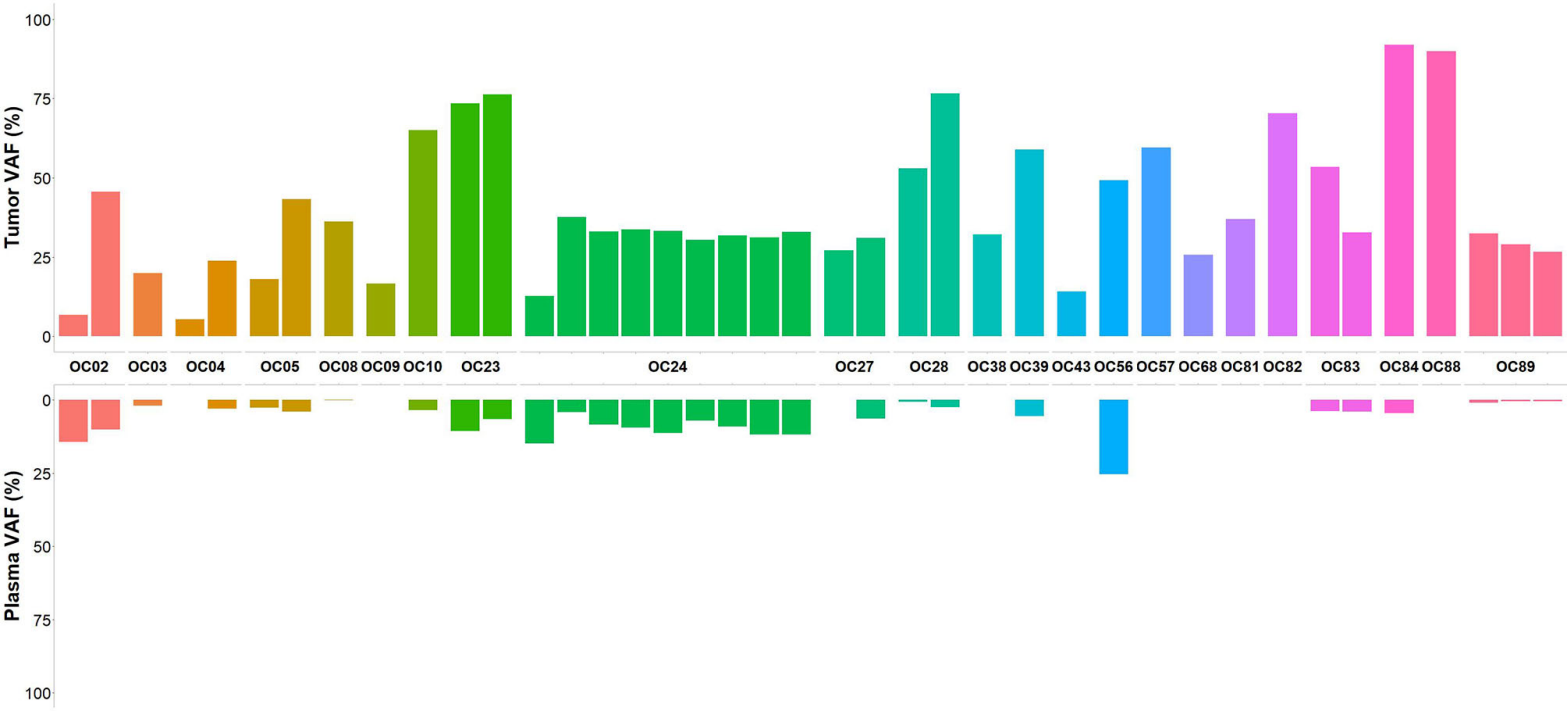
Concordance of variants calls between tumor and plasma samples from the patient treatment naïve group. In patients with more than one somatic variant identified, the VAF for variants shared between matched tumor and plasma samples shows a concordance in hierarchy of VAF in three samples (OC05, OC28 and OC89).

In an attempt to improve the sensitivity to detect somatic variants in plasma, we performed the bioinformatic analysis using the CLC Genomic Workbench, setting 0.2% as the minimum VAF. A total of 21232 variants were annotated and classified among the 96 plasma samples. After variant filtering 14280 variants remained, of which 1328 were classified as pathogenic/likely pathogenic in ClinVar. Among the 12952 variants not classified, 2794 were nonsense, frameshift and variants located at canonical ± 1 or 2 splice sites which were considered pathogenic variants (24). We also retained 1544 inframe deletion and/or insertion variants. The remaining 8614 were missense alterations, of which 2104 were retained as they were predicted to be deleterious by in silico tools. After variant analysis, 7770 variants were identified, of which 37 germline variants were excluded. Thus, 7733 somatic variants were identified among 96 plasma samples. On average, 80 variants were identified in each plasma sample. The overall mutational concordance between variants detected in tumor and matched plasma samples increased to 35.6%. Considering the group of untreated patients, the concordance between tumor and plasma samples for at least one somatic variant was 69.6%. Regarding patients diagnosed with advanced stage disease, at least one somatic variant initially identified in the tumor testing was detected in plasma samples of 83.3% and 61.5% of stage IV and stage III patients, respectively. Among four patients with early-stage disease (stage I), detectable tumor somatic variants were found in plasma samples in three of them (75%). In some patients with more than one shared variant between tumor and plasma sample, the hierarchy of VAF was concordant (OC05, OC28 and OC89) (**Figure 3**). Of the 14 patients with *BRCA1*/*BRCA2* somatic variants identified in tumor samples, two were from the naive treatment group (OC04 and OC28). These patients had a *BRCA1* frameshift somatic variant detected in tumor samples with a VAF of 5.4% and 53%, respectively. In patient OC28 the *BRCA1* somatic variant was also identified in the matched plasma sample at a VAF of 0.7%. Regarding the 67 patients of the post-treatment group, at least one somatic variant present in the tumor was detected in the plasma samples in 22.4% of the patients. In seven of these patients, in whom plasma samples were collected after surgery and before treatment with chemotherapy, somatic variants were detected in 42.9% of plasma samples which belong to three patients who experienced disease relapse and already died. In 12 patients in whom plasma samples were collected at the time of disease recurrence, somatic variants were detected in 33.3% of the plasma samples.

### Accessing Inter- and Intra-Tumor Heterogeneity in cfDNA

**Figure 4:**
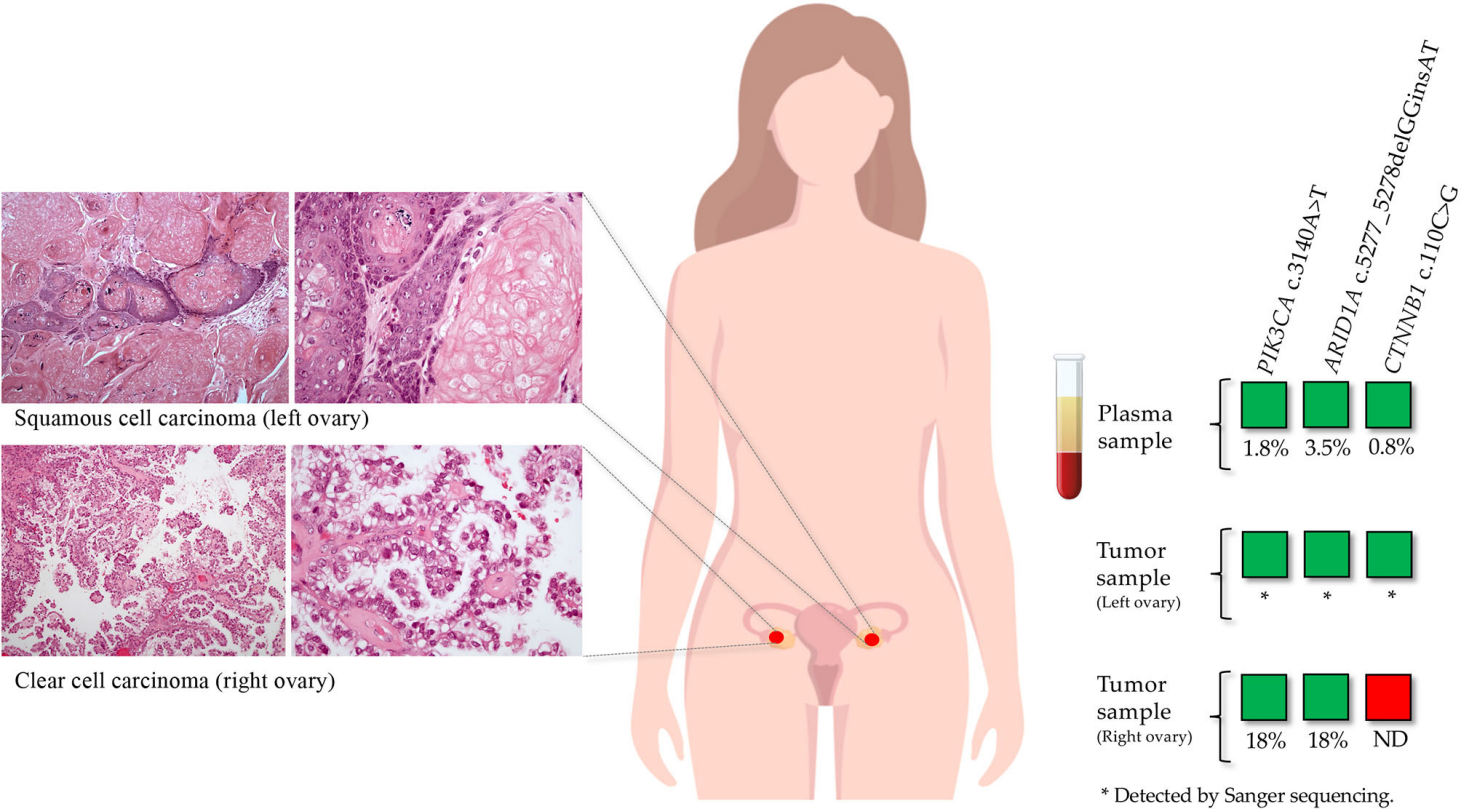
Inter-tumor heterogeneity detected by ctDNA analysis in a patient diagnosed with synchronous tumors on both ovaries. In a tumor sample from the right ovary, a *PIK3CA* and an *ARID1A* pathogenic variants were identified. This tumor consisted of a clear cell carcinoma with a papillary growth pattern. In the matched plasma sample, in addition to these two variants, a *CTNNB1* pathogenic variant was detected. We tested a tumor sample from the left ovary by Sanger sequencing, in which the three variants were identified. The left ovarian tumor consisted of a squamous cell carcinoma with abundant keratinization.

Performing the reverse analysis, in five patients belonging to the treatment naive group we detected pathogenic variants in cfDNA that were not previously identified in the tumor sample. For these patients, FFPE samples from metastasis and/or tumor from a different anatomic location were obtained. In patient OC05, a *CTNNB1* pathogenic variant (c.110C>G) was identified in the plasma sample with a VAF of 0.8%, which was not detected previously in the tumor sample (right ovary; sequence coverage at the variant position: 4762 reads). This variant was further detected in the tumor sample from a different anatomic site (left ovary) in addition to *PIK3CA* and *ARID1A* variants previously identified in the tumor from the right ovary (**Figure 4**). Regarding the other four patients, the variants detected only in the plasma samples were not detected in the analyzed tumor samples from other anatomic sites.

## Discussion

The analysis of ctDNA for tumor molecular profiling represents a potential and attractive alternative to that of FFPE tumor samples for the detection of actionable variants. However, the fraction of ctDNA can be extremely low in a high background of normal cfDNA, so that somatic variants may be present at very low VAF that require extremely sensitive techniques for their detection. Although PCR based methods, such as droplet digital PCR (ddPCR), are very sensitive, they have a low throughput on variant analysis and can only be used to screen for known variants. On the other hand, broader approaches, such as NGS, allow a more complete tumor molecular profiling, enable assessment of inter-tumor and intra-tumor heterogeneity and clonal evolution of the tumor, as well as identification of resistance mechanism to targeted therapy (9, 27–29). Furthermore, recent advances, such as the introduction of unique molecular identifiers (UMIs) and adequate bioinformatic tools, can also help to increase the sensitivity and specificity (30).

The aim of this study was to evaluate the potential of cfDNA to allow detection of somatic tumor driver variants in OC patients, using a NGS approach with a custom gene panel to analyze 96 tumors and matched plasma samples from 96 patients. Initially, the bioinformatic analysis of tumor and plasma samples was performed using GeneGlobe, a web resource for the analysis of Qiagen’s target enrichment panels. However, this is a closed platform that does not allow to define the settings for sequencing alignment and variant calling, including the minimum VAF, which is set at 0.5%. This is suitable for the analysis of FFPE tumor samples as we are looking for variants with a VAF above 5%. However, as somatic variants in ctDNA are present at allele frequencies below the cut-off defined in GeneGlobe, we tested a software that allows to adapt the settings for variant calling in ctDNA analysis. Thus, we reanalyzed plasma samples using CLC Genomics Workbench, which allowed us to lower the minimum VAF to 0.2%, and as expected the overall detection rate of somatic variants in ctDNA improved from 26.7% to 35.6%. Using the most suitable software for ctDNA analysis, at least one somatic variant was detected in ctDNA from 69.6% of the patients from the naive treatment group and in 83.3%, 61.5% and 75% of patients diagnosed with stage IV, stage III and stage I disease, respectively. Furthermore, of the two treatment-naïve patients with somatic *BRCA1* variants identified in FFPE tumor samples, we could detect in ctDNA a *BRCA1* frameshift somatic variant that was present in the tumor sample with a VAF of 53%, but not the one that had a VAF of only 5.4%. These findings support the potential of ctDNA NGS analysis for OC somatic genetic testing, especially when there is no tumor sample available for predictive genetic testing for targeted therapy in untreated stage IV disease. Our results are in agreement with those of a previous study by Phallen and colleagues (31), in which ctDNA alterations were detected in 71% of OC patients, including 75% and 83% of stage III and IV patients. Furthermore, as somatic variants were detected in a great proportion of patients diagnosed with stage I disease, ctDNA analysis also holds the promise to detect early-stage disease, namely as a screening test in healthy women carrying pathogenic germline variants in OC inherited predisposition genes. Recently, Cohen and colleagues (27) reported the development of a blood test based on liquid biopsy that can detect multiple types of cancer at earlier stages, including OC, and which combines the detection of variants in driver genes with the levels of circulating biomarker proteins to detect the presence of a cancer and to determine its origin. The median sensitivity of the test was 73% and 43% for stage II and stage I cancers, respectively.

In addition to noninvasive diagnosis and targeted therapy biomarker identification, ctDNA analysis also holds the promise to be used as a biomarker for prognosis, evaluation of therapy response, monitoring the emergence of resistance during treatment, and disease relapse prediction. This is well exemplified by our finding of somatic variants in ctDNA in the 7 patients belonging to the post-treatment group, from whom plasma samples were collected after surgery and before chemotherapy. All three patients with detectable somatic variants in plasma sample collected after surgery experienced disease relapse (and two of them have already died), while those patients with no detectable somatic variants in plasma after surgery are still disease-free. This suggests that ctDNA analysis can help detect microscopic residual disease after surgery with the potential to be used as prognostic biomarker for OC, allowing the identification of patients at higher risk of recurrence.

One of the advantages of cfDNA is the ability to integrate the detection of somatic alterations from both primary tumor and metastatic lesions, overcoming the problem of intra-tumor and inter-tumor heterogeneity. Our results demonstrated that tumor driver variants detected in ctDNA were not always identified in the FFPE sample initially tested. For instance, in a plasma sample from a patient of the naive treatment group (OC05), in addition to the two variants previously detected in the tumor sample, we detected a *CTNNB1* pathogenic variant with a VAF of 0.8%. This patient was diagnosed with clear cell carcinoma (CCC) of the right ovary (the tumor initially tested with NGS) and poorly differentiated squamous cell carcinoma of the left ovary. To assess the possibility that this discrepancy may be caused by inter-tumor heterogeneity, we analyzed a tumor sample from the left ovary by Sanger sequencing. We confirmed that the *CTNN1B* variant detected in the plasma sample was derived from the tumor of the left ovary, in which we also detected the two variants identified in the tumor from the right ovary. Since these bilateral tumors shared a rare combination of two of the three variants identified in the ctDNA, we could conclude that the two morphologically different tumors were clonally related and not synchronous independent neoplasias, possibly originated from an endometriosis focus that was observed in the periphery of both tumors, which is a well-established precursor lesion of CCC (32). The additional variant found in the tumor of the left ovary probably represents clonal divergence that may at least in part explain the different phenotype of the two tumors (CCC *vs* squamous cell). This observation highlights the ability to detect somatic variants in ctDNA that may be missed by analysis of FFPE samples, confirming that both sample types have complementary roles in managing cancer patients.

The somatic variants not detected in plasma samples may be present with a VAF below 0.2% and would likely be detected using a more sensitive approach. For the detection of variants at VAF as low as <0.2% by NGS, deep sequencing through sequencing the target regions with high coverage (>10,000x) can lower the percentage of false positives (33). Furthermore, most of the cases with no somatic variants detected in ctDNA were those in which only one somatic variant was identified in the tumor sample. Including more genes in the NGS panel could increase the sensitivity, however it would also increase the sequencing costs. In the custom gene panel used in this study, we included genes described in the literature and the COSMIC database as frequently mutated (>10%) in OC. As OC is subclassified in five histological subtypes, which are characterized by different patterns of genetic alterations, it was necessary to include a relatively large number of genes to represent each subtype, resulting in a 27 gene panel. *TP53* variants were highly prevalent in this study (78.0%), as expected given that the majority of FFPE tumor samples were from HGSOC and this tumor type is characterized by *TP53* variants (20). The remaining genes were found mutated in a frequency below 10% because they are associated with the other OC histological subtypes, which are less common and underrepresented in our cohort. Smaller panels specific for each histological subtype would reduce the sequencing costs and would help to improve the sequencing coverage and the sensitivity to detect low frequency variants. On the other hand, since several OC tumor types are relatively rare, it may not be practical to have sequencing runs with only rare histological subtypes. One advantage of using a custom gene panel is the flexibility to manage the genes to include according to the purpose of the study.

Technical and biological factors can impact the concordance between NGS findings in tumor and plasma samples, eventually associated with false-negative and false-positive results for each sample type. False negatives might be explained by the limited amount of ctDNA extracted from plasma samples, which limits the detection of variants with low allele frequency. Weber and colleagues (17), reported that lower DNA input was associated with a decrease of variant calling precision. In our study, the limited amount of DNA extracted from plasma samples (average of 1.3 ng/μL), and consequently a DNA input lower than recommended (average of 17.7 ng), might have been a limitation for the detection of low frequency variants. The DNA input amount influences the number of unique molecular identifiers (UMIs) captured from the original DNA sample, and therefore the sequencing depth, which directly impacts the variant detection sensitivity. False positive results are another challenge when multiple variants are detected by NGS platforms, which has led to the implementation of strategies such as molecular barcodes in order to reduce errors introduced during library preparation (34).

In conclusion, this study demonstrated that somatic variants in genes relevant to OC can be detected in plasma samples of a significant proportion of OC patients, supporting the use of NGS-based ctDNA testing for noninvasive tumor molecular profiling for targeted therapy and to stratify patients according to prognosis.

## Data Availability Statement

The raw data supporting the conclusions of this article will be made available by the authors, without undue reservation.

## Ethics Statement

The studies involving human participants were reviewed and approved by Institutional ethics committee of the Portuguese Oncology Institute of Porto, Portugal. The patients/participants provided their written informed consent to participate in this study.

## Author Contributions

ABa, PP, AP, JG, and MP performed experiments. ABa and PP analyzed data. AP, CB, and RS provided samples. ABa wrote the manuscript. ABa and ABr created the plots. JS performed genetic counseling. MT designed and supervised the study. All authors contributed to the article and approved the submitted version.

## Funding

JG and MP were research fellows of the Fundação para a Ciência e Tecnologia (SFRH/BD/138670/2018 and SFRH/BPD/113014/2015, respectively).

## Conflict of Interest

The authors declare that the research was conducted in the absence of any commercial or financial relationships that could be construed as a potential conflict of interest.

## Publisher’s Note

All claims expressed in this article are solely those of the authors and do not necessarily represent those of their affiliated organizations, or those of the publisher, the editors and the reviewers. Any product that may be evaluated in this article, or claim that may be made by its manufacturer, is not guaranteed or endorsed by the publisher.
